# A study on the correlation between types and duration of moderate-to-vigorous physical activity and mobile phone dependence among adolescents

**DOI:** 10.3389/fpubh.2026.1870655

**Published:** 2026-08-21

**Authors:** Meiqi Yang

**Affiliations:** School of Competitive Sport, Beijing Sport University, Beijing, China

**Keywords:** adolescents, closed-skill, mobile phone dependence, moderate-to-vigorous physical activity, open-skill

## Abstract

**Objective:**

To explore the correlation between the type and duration of moderate-to- vigorous physical activity (MVPA) and mobile phone dependence among adolescents, so as to provide a scientific basis for intervention strategies for mobile phone dependence.

**Methods:**

A total of 971 eighth-grade junior high school students in Dalian were selected by mixed sampling. The Chinese version of the Children’s Leisure Activities Study Survey was used to measure the type and duration of MVPA, and the Adolescent Mobile Phone Dependence Scale was used to assess the level of mobile phone dependence. Data were analyzed using independent samples *t*-test, generalized linear model, and stratified regression analysis.

**Results:**

Open-skill MVPA was significantly negatively correlated with physical and mental impacts caused by mobile phone dependence (*β* = −0.777, 95%*CI*: −1.268 to −0.287, *p* < 0.01). Meeting the MVPA duration standard was significantly positively correlated with withdrawal symptoms of mobile phone dependence (*β* = 1.469, 95%*CI*: 0.574 to 2.364, *p* < 0.01). Among participants in open-skill PA, the negative association between meeting the duration standard and withdrawal symptoms was stronger (*β* = −1.890, 95%*CI*: −3.046 to −0.733, *p* < 0.01). By contrast, closed-skill PA showed no significant correlation with any dimension of mobile phone dependence, regardless of whether the duration standard was met (all *p* > 0.05).

**Conclusion:**

Simply meeting the daily recommended duration of MVPA fails to alleviate adolescents’ mobile phone withdrawal symptoms; only combining sufficient MVPA duration with open-skill PA yields a protective effect against mobile phone dependence. The findings of this study can provide a reference for PA arrangements in primary and secondary schools. It is recommended to promote open-skill PA and ensure that students meet the daily recommended duration of MVPA. Nevertheless, all data in this study were collected via self-reported questionnaires. Future research may integrate objective monitoring tools to further verify the findings of the present study.

## 1 Introduction

Mobile phone dependence refers to individuals’ strong psychological and behavioral dependence on mobile phone use, characterized by uncontrollable usage urges and excessive use that impairs daily life and study (1, 2). Adolescents are in a critical period of physical and mental development, and mobile phone dependence may exert various negative impacts on their academic performance, interpersonal communication, physical and mental health (2, 3). Therefore, exploring effective interventions for adolescent mobile phone dependence holds important practical significance.

Physical activity (PA), as an active lifestyle, has been widely proven to confer benefits on the physical and mental health of adolescents (4–7). The World Health Organization (WHO) recommends that adolescents accumulate at least 60 min of moderate-to-vigorous physical activity (MVPA) per day on average each week (8). Relevant studies (9–11) have found an association between PA and mobile phone dependence in adolescents. Sagre & Ahlawat (12) found through a survey that students’ PA level was negatively correlated with mobile phone dependence, namely higher PA was linked to milder mobile phone dependence. Ren et al. (13) confirmed via comparative analysis that adolescents without smartphone addiction spent significantly more time on MVPA. Hu et al. (14) further indicated that adolescents who met the WHO recommendations for MVPA had a lower overall level of mobile phone dependence.

In addition, according to the motor skill classification theory, PA is categorized into open-skill PA and closed-skill PA (15). Centered on the predictability of the external environment during movement execution, this classification framework is well supported by established theories in sport psychology (16). Open-skill PA refers to movements that are highly dependent on a dynamically changing external environment, requiring individuals to perceive, judge and adjust their motions in real time (16). Closed-skill PA describes movements largely unaffected by external environmental disturbances, where individuals repeatedly perform standardized actions within a stable and predictable setting (16). Within this classification framework, Zhang et al. (17) found that adolescents engaging in open-skill PA exhibited lower levels of mobile phone dependence than those participating in closed-skill PA. In terms of the underlying mechanism, open-skill PA constantly demands individuals to dynamically perceive surroundings and rapidly adjust movements, which persistently activates executive functions and improves adolescents’ self-control (18). Furthermore, open-skill PA involves extensive peer cooperation and real-time interpersonal interaction (19). It can sufficiently satisfy adolescents’ social needs, alleviate loneliness and anxiety, and reduce their behavioral motivation to rely on mobile phones as an escape from negative emotions (19).

However, existing research still has some limitations. Existing relevant studies tend to separate the two core dimensions of MVPA, namely activity type and duration. Most only explore the correlation between either duration or type and mobile phone dependence in isolation, while lacking analyses on their joint interactive effects. Theoretical analysis indicates that type determines the cognitive stimulation and social interactive attributes of PA, whereas duration defines the cumulative dose of behavioral intervention. These two dimensions constitute an interdependent and inseparable organic whole. Analyzing only a single dimension in isolation cannot fully uncover the genuine association between MVPA and mobile phone dependence. In reality, PA among adolescents is an organic combination of type, intensity, and duration. However, research on their joint effects remains scarce, making it difficult for educators and parents to reasonably arrange PA to intervene in adolescents’ mobile phone dependence in a targeted manner.

Accordingly, this study comprehensively incorporates the type, intensity, and duration of PA, and examines the combined association between type and duration of MVPA and mobile phone dependence among adolescents, providing a scientific basis for establishing PA guidelines and intervention programs for adolescent mobile phone dependence.

## 2 Methods

### 2.1 Participants

This study calculated the sample size based on the principle of 10 ~ 20 times the number of independent variables. With 9 independent variables included, 90 ~ 180 valid samples were required. Allowing for a 20% invalid data rate, a total of 216 samples was needed. A mixed sampling method was adopted. First, one junior high school was conveniently selected from each of the four districts (Zhongshan, Xigang, Shahekou, and Ganzi) in Dalian City, Liaoning Province, China. Second, six Grades 8 classes were randomly chosen from each school for investigation. The survey population consisted of 1,091 students from 24 classes.

Grade 8 students were selected for the following reasons: Grade 7 students had just entered junior school and had not fully adapted to the school routine and study rhythm; their PA habits and mobile phone use behaviors were unstable, leading to large data fluctuations. Grade 9 students faced intense pressure from the senior school entrance examination, with heavy academic burdens and limited time, which severely restricted their PA and mobile phone use and failed to reflect normal conditions. In contrast, Grade 8 students had adapted to junior school life, experienced moderate academic pressure, and were in a stable stage of physical and mental development. Their PA patterns and mobile phone dependence were more representative and stable, allowing for a truer reflection of the relationships between variables.

**Figure 1:**
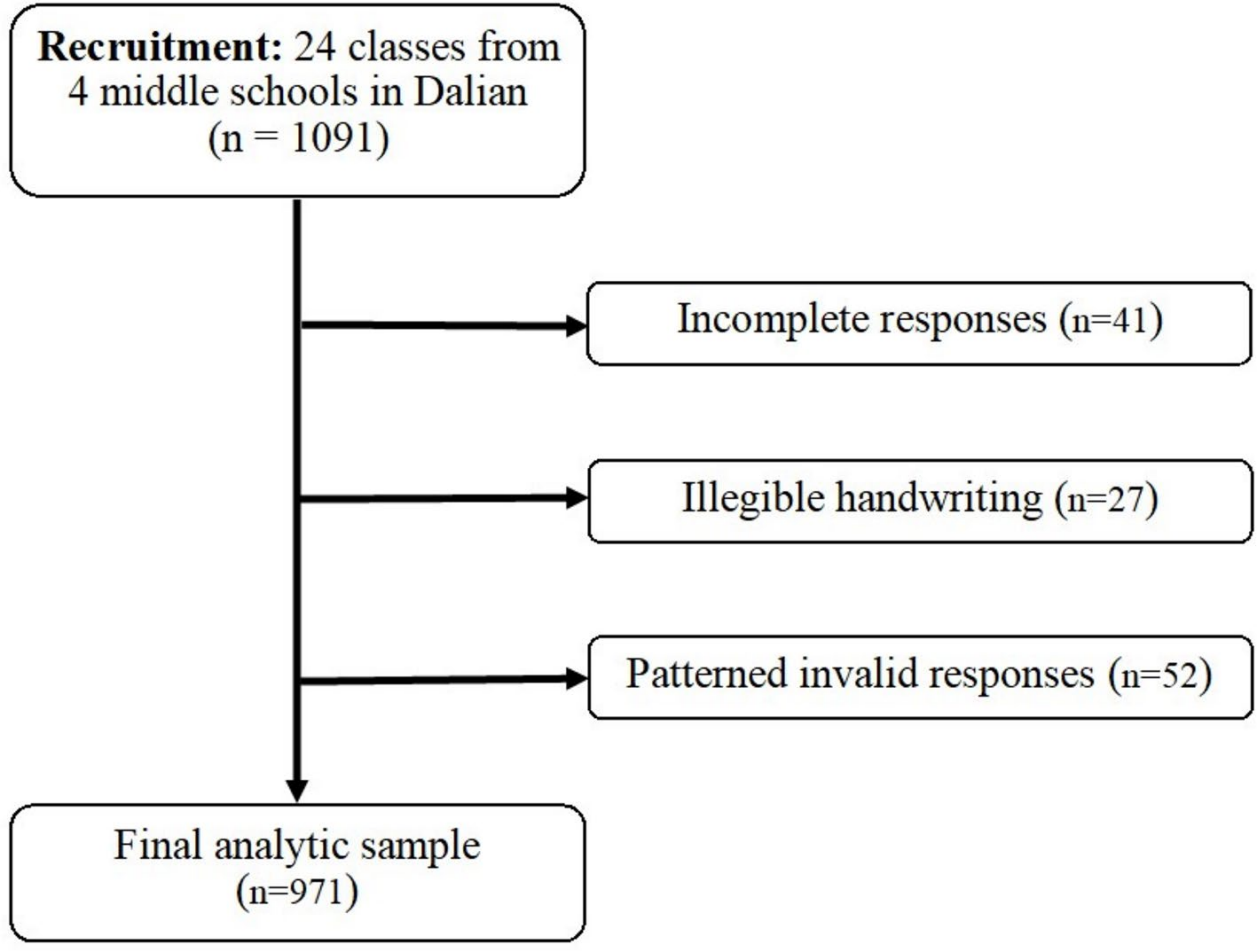
Participant flow diagram.

During data sorting, a total of 120 invalid questionnaires were excluded. The invalid responses fell into three main categories: incomplete questionnaires with a large number of unanswered items, illegible handwritten answers, and patterned random responses, such as selecting the same option for all questions or filling in answers following a fixed Z-shaped pattern. After removing these invalid data, a final sample of 971 students was included for subsequent data analysis (see Figure 1). This study was approved by the Ethics Committee of Sports Science Experiments, Beijing Sport University (Approval No.: 2026201H), and written informed consent was obtained from all participants and their guardians.

### 2.2 Variables and measures

#### 2.2.1 Type and duration of MVPA

This study adopted the Chinese version of the Children’s Leisure Activities Study Survey (CLASS-C) to assess the type and duration of students’ MVPA. The instrument was originally developed by Telford et al. (20) and later revised by Huang et al. (21) and Li et al. (22) to adapt to the context of Chinese adolescents, demonstrating satisfactory reliability and validity. This study examined the internal consistency reliability of the scale among 971 valid participants. The Cronbach’s *α* coefficient was 0.821, indicating good internal consistency reliability. The questionnaire covers the frequency and duration of 25 types of PA, including soccer, basketball, gymnastics, and others.

In this study, PA types were categorized into open-skill PA and closed-skill PA based on the predictability of environmental changes during motor tasks (23). Open-skill PA refers to activities performed in unpredictable environments, where individuals must continuously respond and adjust movements in real time to environmental changes; typical examples include soccer, basketball, table tennis, tennis, and taekwondo. Closed-skill PA refers to activities conducted in stable and predictable environments, where individuals can preplan complete movement programs prior to participation; typical examples include dance, artistic gymnastics, martial arts routines, and track and field events. Existing studies on exercise interventions for adolescents classify dominant PA based on the type of sport that accounts for the largest proportion of daily participation duration. For instance, relevant systematic reviews and meta-analyses (18, 23, 24) define comprehensive PA interventions with open-skill characteristics as open-skill PA-dominant interventions. Furthermore, Chinese adolescents have limited spare time for PA after school, and sporadic engagement in other sports cannot sustainably reshape their cognitive and social functioning (25). Accordingly, this classification approach aligns with the present study’s objective of exploring the association between long-term sport types and mobile phone dependence. Accordingly, participants were classified into the open-skill PA group and the closed-skill PA group according to the time spent in open-skill and closed-skill PA.

Furthermore, weekly accumulated PA time was used to measure students’ PA levels. PA intensity was assessed via self-perceived exertion, where “somewhat tired” and “very tired” represented moderate physical activity (MPA) and vigorous physical activity (VPA), respectively. Thus, MVPA duration was calculated as the sum of MPA and VPA duration.

#### 2.2.2 Mobile phone dependence

In this study, the Adolescent Mobile Phone Dependence Self-Rating Questionnaire developed by Tao et al. (26) was used to assess mobile phone dependence among primary school students. The questionnaire consists of 13 items covering three dimensions: withdrawal symptoms (6 items), craving (3 items), and physical and mental impacts (4 items). The questionnaire employs a 5-point Likert self-rating scale, with a total score ranging from 13 to 65. Higher scores indicate more severe mobile phone dependence, and a score of 27 or higher is classified as mobile phone dependence. Previous research (26) confirmed that the Cronbach’s *α* coefficient for the full questionnaire was 0.87, and the Cronbach’s *α* coefficients for each dimension ranged from 0.58 to 0.83, indicating satisfactory internal consistency reliability. This study examined the internal consistency reliability of the scale among 971 valid participants. The Cronbach’s α coefficient was 0.809, indicating good internal consistency reliability.

#### 2.2.3 Control variables

Referring to existing studies on the influencing factors of smartphone addiction (27–29), this study controlled for variables including age, gender, residential area, body mass index (BMI), and socioeconomic status (SES). Residential area was categorized as urban or rural. BMI was calculated based on height and weight using the formula: BMI = kg/m^2^. The MacArthur Subjective Socioeconomic Status Scale (30) was adapted to measure adolescents’ SES. Composed of a ladder figure and related questions, the scale includes 10 rungs, with the top rung representing people with the highest SES and the bottom rung representing those with the lowest SES. Adolescents placed themselves on one of the rungs according to their subjective perceptions to indicate their subjective SES. Previous studies (31, 32) have verified that this scale can effectively evaluate respondents’ SES and has been widely used in large-scale surveys such as the Chinese General Social Survey.

### 2.3 Data analysis

SPSS 25.0 was used for data processing and statistical analysis in this study. Descriptive statistics for continuous variables were presented as mean (*M*) and standard deviation (*SD*), while those for categorical variables were expressed as frequencies and percentages. First, the one-sample Kolmogorov–Smirnov test combined with P–P and Q-Q plots was conducted to test the normality of mobile phone dependence data, which showed an approximately normal distribution. Thus, the independent samples *t*-test was adopted to perform intergroup comparative analyses based on demographic characteristics including gender and residential area, as well as PA-related information such as PA type and compliance with the MVPA guidelines. Second, this study adopted the linear response model based on the generalized linear model (GLM). We set PA type and adherence to the MVPA guideline as the independent variables, and mobile phone dependence as the dependent variable. We investigated the associations of PA type and MVPA guideline adherence with mobile phone dependence among adolescents after adjusting for relevant confounding factors. The Wald *χ*^2^ statistic was used to test the model effects. Finally, with PA type as the stratification variable, the association between the combination of PA type and compliance with the MVPA guidelines and adolescent mobile phone dependence was investigated. In addition, to reduce the potential risk of Type I error brought by subgroup analyses, this study adopted a large-sample survey design with balanced sample sizes across the two subgroups to improve statistical stability, and a consistent stringent significance level of *α* = 0.05 was applied throughout all tests. Through the above sequential research, the association characteristics between MVPA and adolescent mobile phone dependence were comprehensively revealed.

## 3 Results

### 3.1 Basic information of participants

The average age of the participants was 13.09 years (*SD* = 0.54), with an average BMI of 21.55 (*SD* = 5.07), a mean socioeconomic status of 5.78 (*SD* = 0.91), and a mean moderate-to-vigorous physical activity of 245.11 (*SD* = 289.78). The mean scores for withdrawal symptoms, craving, and physical and mental impacts of smartphone addiction were 13.27, 6.77, and 9.70, respectively. In terms of physical activity types, open physical activity accounted for 57.2% (*n* = 555), and closed physical activity accounted for 42.8% (*n* = 416). A total of 81.6% (*n* = 792) of the participants met the MVPA guidelines, whereas 18.4% (*n* = 179) did not. Regarding gender distribution, males accounted for 38.2% (*n* = 371) and females accounted for 61.8% (*n* = 600). In terms of residential area, 24.7% (*n* = 240) of the participants were urban students and 75.3% (*n* = 731) were rural students. Overall, the sample of this study was predominantly composed of rural students and female adolescents. The basic information of the participants is shown in Table 1.

**Table 1:** Basic information of participants.

Variables	*M*	*SD*
Age	13.09	0.54
BMI	21.55	5.07
SES	5.78	0.91
MVPA	245.11	289.78
Withdrawal symptoms	13.27	5.65
Craving	6.77	2.69
Physical and mental impacts	9.70	3.79

Variable	Freq.	%
PA types		
Open	555	57.2
Closed	416	42.8
MVPA guidelines		
Meet	792	81.6
Not meet	179	18.4
Gender		
Male	371	38.2
Female	600	61.8
Residential area		
Urban	240	24.7
Rural	731	75.3

### 3.2 Comparative analysis of adolescent mobile phone dependence across groups

#### 3.2.1 Comparative analysis of adolescent mobile phone dependence by gender and residential area

**Figure 2:**
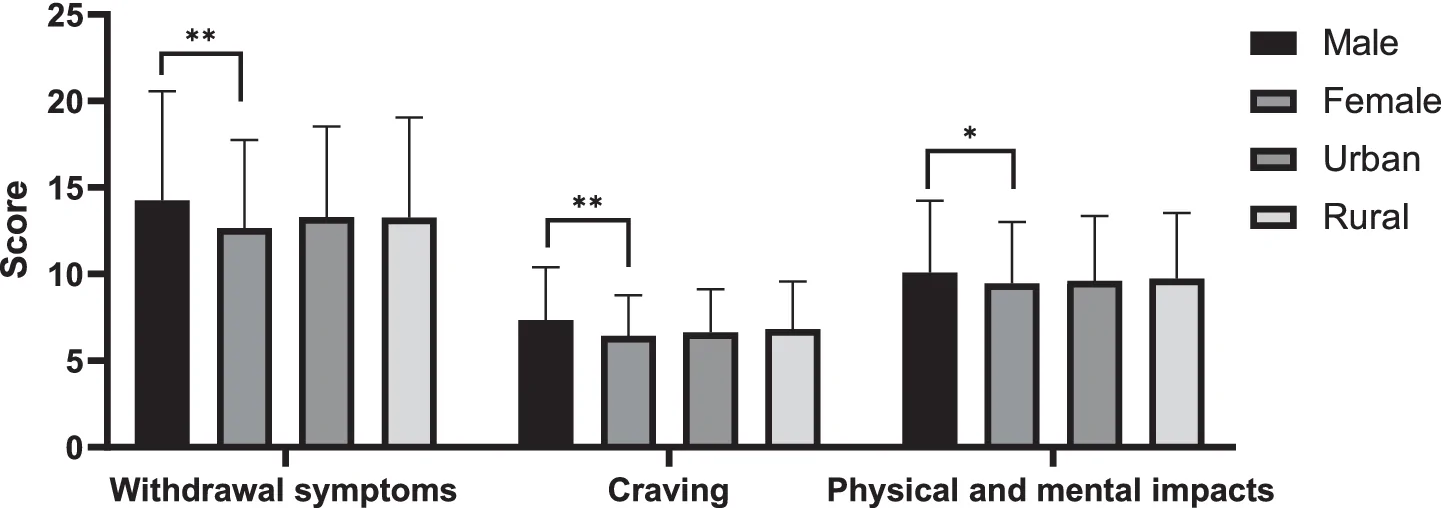
Results of intergroup comparative analysis of adolescent mobile phone dependence in terms of gender and residential area (*p < 0.05; **p < 0.01).

The results of the intergroup comparative analysis of adolescent mobile phone dependence by gender (Figure 2) showed that male adolescents had significantly higher withdrawal symptoms (*t* = 4.119, *p* < 0.001), craving (*t* = 4.980, *p* < 0.001), and physical and mental impacts (*t* = 2.464, *p* = 0.014) than female adolescents. The results of the intergroup comparative analysis of adolescent mobile phone dependence by place of residence (Figure 2) indicated that there were no significant differences in withdrawal symptoms, craving, or physical and mental impacts between urban and rural adolescents (*p* > 0.05).

#### 3.2.2 Comparative analysis of adolescent mobile phone dependence by PA types

**Figure 3:**
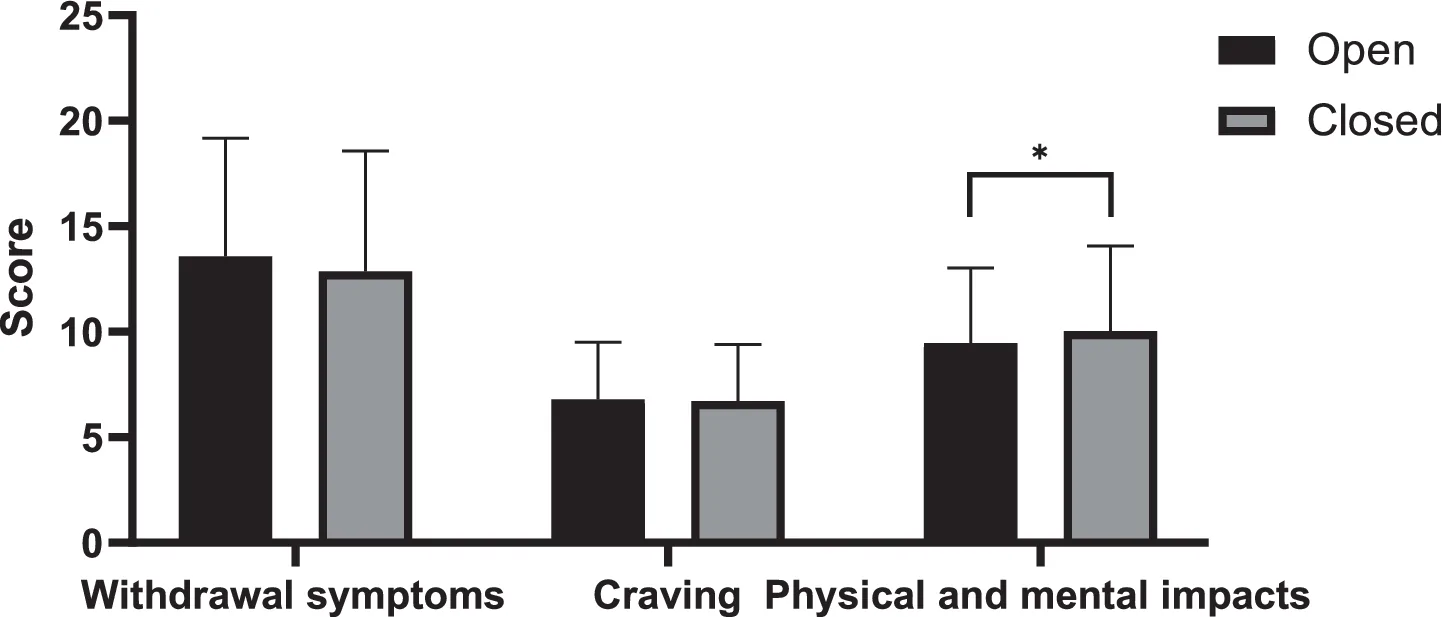
Results of intergroup comparative analysis of adolescent mobile phone dependence in terms of PA types (*p < 0.05).

The results of the intergroup comparative analysis of adolescent mobile phone dependence by PA types (Figure 3) showed that adolescents engaging in PA dominated by open motor skills had significantly lower physical and mental impacts than those participating in PA dominated by closed motor skills (*t* = −2.283, *p* = 0.023). However, there were no significant differences between the two groups in terms of withdrawal symptoms and craving (*p* > 0.05).

#### 3.2.3 Comparative analysis of adolescent mobile phone dependence by MVPA guidelines

**Figure 4:**
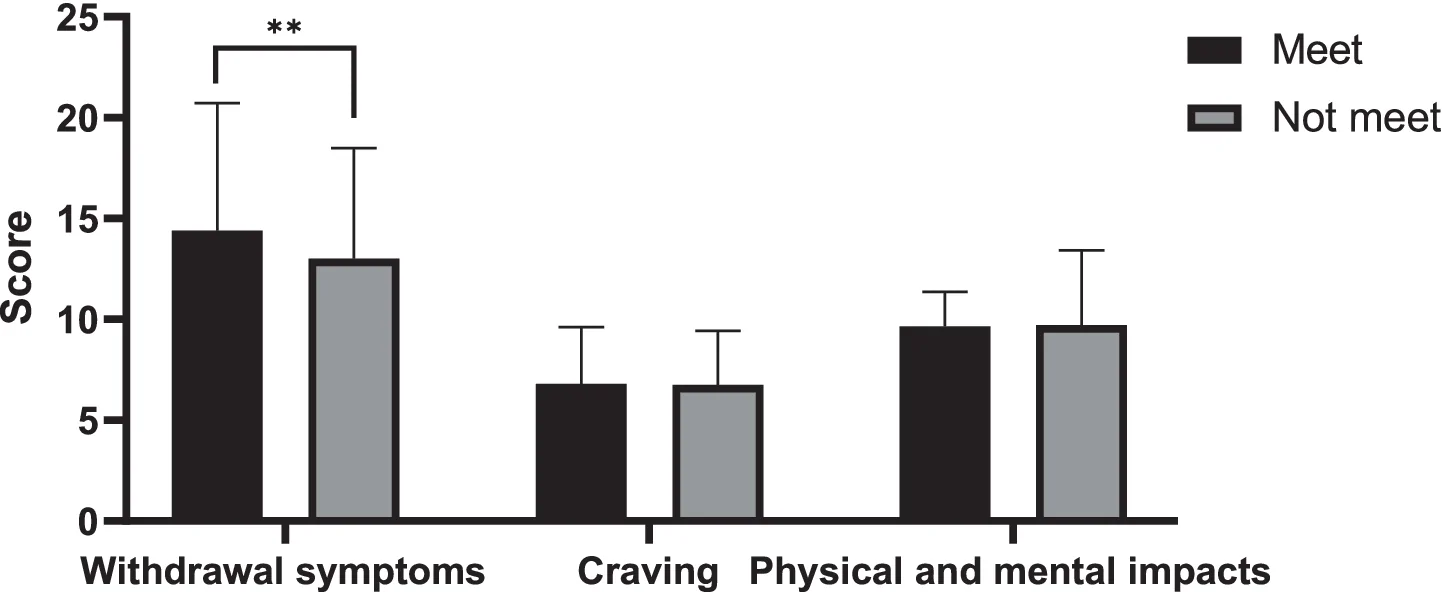
Results of intergroup comparative analysis of adolescent mobile phone dependence based on whether they meet MVPA guidelines (**p < 0.01).

The results of the intergroup comparative analysis of adolescent mobile phone dependence based on whether they meet the MVPA guidelines (Figure 4) showed that adolescents who meet the MVPA guidelines had significantly higher withdrawal symptoms than those who do not (*t* = −2.722, *p* = 0.007). However, there were no significant differences between the two groups in terms of craving and physical and mental impacts (*p* > 0.05).

### 3.3 Correlation between PA types and adolescents’ mobile phone dependence

Results of the multiple linear regression analysis on the relationship between PA types and adolescents’ mobile phone dependence (Table 2) showed that there was no significant correlation between PA types and withdrawal symptoms caused by mobile phone dependence (*β* = 0.310, 95%*CI* = -0.416 ~ 1.035, Wald *χ*^2^ = 0.710, *p* = 0.403) or craving (*β* = −0.046, 95%*CI* = -0.390 ~ 0.298, Wald *χ*^2^ = 0.069, *p* = 0.792) among adolescents. However, the adolescent group who frequently participated in open-skill MVPA had significantly lower physical and mental impacts caused by mobile phone dependence than those who engaged in closed-skill MVPA (*β* = −0.777, 95%*CI* = −1.268 ~ 0.287, Wald *χ*^2^ = 9.654, *p* = 0.002). Regarding relevant control variables, age was significantly positively correlated with withdrawal symptoms, craving, and physical and mental impacts (*p* < 0.05); male adolescents had significantly higher withdrawal symptoms, craving, and physical and mental impacts than females (*p* < 0.01); SES was significantly negatively correlated with craving (*p* < 0.01); while the correlations between residence, BMI and adolescents’ mobile phone dependence were not significant (*p* > 0.05).

**Table 2:** Results of multiple linear regression analysis on the correlation between PA types and adolescents’ mobile phone dependence.

Variables	Withdrawal symptoms		Craving		Physical and mental impacts	
	*β*	95%*CI*	*β*	95%*CI*	*β*	95%*CI*
PA types (Open)	0.310	(−0.416, 1.035)	−0.046	(−0.390, 0.298)	−0.777**	(−1.268, −0.287)
Age	1.349**	(0.700, 1.999)	0.613**	(0.306, 0.921)	0.512*	(0.073, 0.951)
Gender (Male)	1.560**	(0.817, 2.303)	0.833**	(0.481, 1.185)	0.818**	(0.316, 1.320)
Residential area (Rural)	0.229	(−0.592, 1.050)	−0.051	(−0.440, 0.338)	−0.135	(−0.690, 0.420)
BMI	−0.030	(−0.100, 0.040)	0.020	(−0.013, 0.054)	0.013	(−0.034, 0.060)
SES	0.272	(−0.124, 0.667)	−0.225*	(−0.413, −0.038)	0.238	(−0.030, 0.505)

*p < 0.05; **p < 0.01.

### 3.4 Correlation between meeting the MVPA guidelines and adolescents’ mobile phone dependence

Table 3 shows the results of multiple linear regression analysis on the relationship between meeting the MVPA guidelines and mobile phone dependence among adolescents. Compared with the adolescent group who did not meet the MVPA guidelines, the adolescent group who met the MVPA guidelines had higher withdrawal symptoms (*β* = 1.469, 95%*CI* = 0.574 ~ 2.364, Wald *χ*^2^ = 10.356, *p* = 0.001); however, there were no significant differences in craving (*β* = −0.026, 95%*CI* = −0.452 ~ 0.400, Wald *χ*^2^ = 0.015, *p* = 0.904) and physical and mental impacts (*β* = 0.025, 95%*CI* = -0.586 ~ 0.636, Wald *χ*^2^ = 0.006, *p* = 0.936) between the adolescent groups who met and did not meet the MVPA guidelines. In terms of relevant control variables, age was not significantly correlated with withdrawal symptoms, craving and physical and mental impacts caused by mobile phone dependence (*p* < 0.05); male adolescents had significantly higher withdrawal symptoms and craving caused by mobile phone dependence than female adolescents (*p* < 0.01); SES was significantly negatively correlated with craving caused by mobile phone dependence (*p* < 0.05); while residential area and BMI were not significantly correlated with mobile phone dependence (*p* > 0.05).

**Table 3:** Results of multiple linear regression analysis on the correlation between meeting the MVPA guidelines and adolescents’ mobile phone dependence.

Variables	Withdrawal symptoms		Craving		Physical and mental impacts	
	*β*	95%*CI*	*β*	95%*CI*	*β*	95%*CI*
MVPA guidelines (Meet)	1.469**	(0.574, 2.364)	−0.026	(−0.453, 0.400)	0.025	(−0.586, 0.636)
Age	1.327**	(0.680, 1.973)	0.613**	(0.305, 0.921)	0.511*	(0.069, 0.952)
Gender (Male)	1.674**	(0.952, 2.396)	0.824**	(0.480, 1.168)	0.645*	(0.152, 1.138)
Residential area (Rural)	0.213	(−0.604, 1.030)	−0.049	(−0.438, 0.340)	−0.102	(−0.660, 0.455)
BMI	−0.035	(−0.104, 0.035)	0.021	(−0.012, 0.054)	0.019	(−0.029, 0.066)
SES	0.325	(−0.067, 0.716)	−0.227*	(−0.414, −0.041)	0.190	(−0.077, 0.458)

*p < 0.05; **p < 0.01.

### 3.5 Correlation between the combination of PA types and MVPA guidelines and mobile phone dependence among adolescents

#### 3.5.1 Correlation between meeting the open-skill MVPA guidelines and mobile phone dependence among adolescents

The results of the correlation analysis between meeting the guidelines for open-skill MVPA and mobile phone dependence among adolescents (Table 4) showed that compared with the adolescent group not meeting the guidelines for open-skill MVPA, the group meeting these guidelines had significantly lower withdrawal symptoms (*β* = −1.890, 95%*CI* = −3.046 ~ −0.733, Wald *χ*^2^ = 10.261, *p* = 0.001). There were no significant differences in craving (*β* = 0.133, 95%*CI* = −0.424 ~ 0.690, Wald *χ*^2^ = 0.218, *p* = 0.640) and physical and mental impacts (*β* = −0.208, 95%*CI* = −0.964 ~ 0.548, Wald *χ*^2^ = 0.292, *p* = 0.589) between the two groups. Regarding relevant control variables, age was significantly positively correlated with withdrawal symptoms and craving (*p* < 0.01); male adolescents had significantly higher withdrawal symptoms and craving than female adolescents (*p* < 0.01); BMI was significantly negatively correlated with withdrawal symptoms (*p* < 0.05); SES was significantly negatively correlated with craving (*p* < 0.05); while the correlation between residential area and adolescents’ mobile phone dependence was not significant (*p* > 0.05).

**Table 4:** Results of multiple linear regression analysis on the correlation between meeting the open-skill MVPA guidelines and mobile phone dependence among adolescents.

Variables	Withdrawal symptoms		Craving		Physical and mental impacts	
	*β*	95%*CI*	*β*	95%*CI*	*β*	95%*CI*
MVPA guidelines (Meet)	−1.890**	(−3.046, −0.733)	0.133	(−0.424, 0.690)	−0.208	(−0.964, 0.548)
Age	1.559**	(0.698, 2.420)	0.741**	(0.326, 1.156)	0.465	(−0.098, 1.028)
Gender (Male)	1.521**	(0.596, 2.446)	0.798**	(0.352, 1.243)	0.561	(−0.044, 1.166)
Residential area (Rural)	−0.230	(−1.331, 0.871)	−0.388	(−0.919, 0.142)	−0.516	(−1.236, 0.204)
BMI	−0.123*	(−0.220, −0.025)	−0.034	(−0.081, 0.013)	−0.040	(−0.104, 0.024)
SES	0.024	(−0.549, 0.598)	−0.306*	(−0.582, −0.029)	0.090	(−0.285, 0.465)

*p < 0.05; **p < 0.01.

#### 3.5.2 Correlation between meeting the closed-skill MVPA guidelines and mobile phone dependence among adolescents

The results of multiple linear regression analysis on meeting the closed-skill MVPA guidelines and adolescent mobile phone dependence (Table 5) showed that there were no significant differences in withdrawal symptoms (*β* = −0.663, 95%*CI* = −2.076 ~ 0.750, Wald *χ*^2^ = 0.846, *p* = 0.358), craving (*β* = −0.210, 95%*CI* = -0.868 ~ 0.449, Wald *χ*^2^ = 0.390, *p* = 0.533), and physical and mental impacts (*β* = 0.381, 95%*CI* = −0.624 ~ 1.386, Wald *χ*^2^ = 0.552, *p* = 0.457) between adolescents who met the closed-skill MVPA guidelines and those who did not. Regarding relevant control variables, male adolescents had significantly higher scores in withdrawal symptoms, craving, and physical and mental impacts than female adolescents (*p* < 0.01); BMI was significantly positively correlated with craving and physical and mental impacts (*p* < 0.05); SES was significantly positively correlated with withdrawal symptoms and physical and mental impacts (*p* < 0.05); however, age and place of residence had no significant correlation with adolescent mobile phone dependence (*p* > 0.05).

**Table 5:** Results of multiple linear regression analysis on the correlation between meeting the closed-skill MVPA guidelines and mobile phone dependence among adolescents.

Variables	Withdrawal symptoms		Craving		Physical and mental impacts	
	*β*	95%*CI*	*β*	95%*CI*	*β*	95%*CI*
MVPA guidelines (Meet)	−0.663	(−2.076, 0.750)	−0.210	(−0.868, 0.449)	0.381	(−0.624, 1.386)
Age	0.761	(−0.235, 1.757)	0.378	(−0.086, 0.842)	0.374	(−0.334, 1.083)
Gender (Male)	2.412**	(1.162, 3.661)	1.163**	(0.581, 1.746)	1.604**	(0.715, 2.493)
Residential area (Rural)	1.051	(−0.178, 2.280)	0.515	(−0.058, 1.088)	0.462	(−0.412, 1.337)
BMI	0.064	(−0.036, 0.163)	0.080**	(0.033, 0.126)	0.078*	(0.007, 0.149)
SES	0.609*	(0.064, 1.154)	−0.151	(−0.405, 0.103)	0.395*	(0.007, 0.782)

*p < 0.05; **p < 0.01.

## 4 Discussions

### 4.1 Adolescents who engage in open-skill MVPA have lower levels of mobile phone dependence

The study found that adolescents participating in open-skill MVPA exhibited significantly lower levels of mobile phone dependence compared with those engaged in closed-skill MVPA. The differences were mainly reflected in the physical and psychological impairment dimension caused by mobile phone dependence. The findings of this study are consistent with those reported by Zhang et al. (17), further confirming that open-skill MVPA has a significantly stronger negative correlation with smartphone addiction among adolescents than closed-skill MVPA.

From the perspective of activity characteristics, open-skill MVPA is characterized by interaction and cooperation, variable situations, real-time decision-making and interpersonal communication (33, 34). During exercise, continuous peer communication, tactical cooperation and dynamic environmental judgment are required, which fully activates adolescents’ social needs and occupies cognitive resources (35, 36), effectively filling fragmented spare time and reducing incentives for mindless mobile phone scrolling. In addition, frequent interpersonal interaction alleviates negative emotions such as loneliness and anxiety (37), reducing adolescents’ tendency to rely on mobile phones to escape emotional pressure at the psychological level (38, 39). In contrast, closed-skill MVPA mostly involves individual practice with fixed exercise scenarios and insufficient social interaction (33, 34). It only satisfies emotional regulation and social needs to a limited extent and thus cannot form a strong buffering effect against mobile phone dependence (40).

In addition, executive function is closely associated with mobile phone dependence (41–43). Conducted in dynamic and unpredictable environments, open-skill MVPA requires participants to perceive environmental changes in real time, make rapid judgments and adjust motor strategies. This process fully activates brain executive functions (16, 23). Long-term participation in such activities improves adolescents’ cognitive regulation and environmental adaptation abilities, enabling them to break away from dependence on the virtual mobile environment and reduce the physical and mental disturbances caused by mobile phone use (17). By comparison, closed-skill MVPA is performed in stable and predictable settings with standardized, repetitive movements and low demands for real-time cognitive adjustment (41–43). While it can improve physiological indicators such as cardiopulmonary function, it exerts limited activation on cognitive function (24), resulting in a weak negative correlation with the physical and mental impacts caused by mobile phone dependence.

In terms of physical and psychological differences, the social connection and emotional empowerment brought by open-skill MVPA can directly ameliorate physical and mental problems caused by excessive mobile phone use, including sleep disturbance, irritability and fatigue (44). Unlike closed-skill MVPA, which merely promotes physical fitness, open-skill MVPA integrates physical health maintenance, social support and emotional regulation (45, 46). It comprehensively mitigates the adverse physical and mental effects of mobile phone dependence among adolescents, which explains the significant intergroup differences observed in the physical and psychological dimension. Combined with existing research consensus and the findings of this study, it can be further inferred that the social attributes and situational complexity of PA may be key variables leading to inconsistent correlations.

### 4.2 Adolescents who meet the MVPA guidelines exhibit higher mobile phone dependence

This study found that adolescents who met the MVPA guidelines exhibited higher levels of mobile phone dependence than those who did not, with a more pronounced difference in the dimension of withdrawal symptoms; however, no significant differences were observed in the dimensions of craving and physical and mental impacts. This result is contrary to previous studies (10, 14). Combined with the study design, variable characteristics, and sample features, it can be discussed from four perspectives: behavioral mechanism, sample characteristics, and measurement logic.

Firstly, adolescents may engage in compensatory smartphone use during rest periods after completing MVPA. That is, being restricted from using mobile phones during MVPA may lead to a stronger urge for subsequent use. This phenomenon is quite common in daily life. For instance, after a high-intensity physical education class featuring basketball, soccer, students immediately start scrolling through short videos, chatting or playing games as soon as they return to the classroom or dormitory. Similarly, teenagers who go running or attend ball sports training sessions on weekends will pick up their phones right after the activity and spend a long time on them. This pattern of suppressed phone use followed by concentrated indulgence significantly intensifies the withdrawal symptoms of mobile phone dependence. In addition, this study is a cross-sectional design that only reflects contemporaneous associations: adolescents meeting the MVPA guidelines have reduced leisure time available for smartphone use due to physical activity, which amplifies withdrawal symptoms; those not meeting the guidelines spend more time in sedentary behaviors and use smartphones more continuously, so their dependent behaviors may remain in a satiated state.

Secondly, the participants in this study were eighth-grade students, who experience moderate academic pressure but have already formed stable smartphone use habits. Adolescents who consistently engage in adequate MVPA generally possess strong self-management skills, or study in classes and schools with strict sports and mobile phone regulations. Subject to long-term restrictions on mobile phone use at school, they tend to develop an intense craving for phones. Once away from such regulated environments, their withdrawal symptoms become prominent. By contrast, adolescents not meeting the MVPA guidelines tend to prefer sedentary behaviors and lack supervision for physical activity; their smartphone use is unrestricted, and dependence remains in a stable manifestation state, resulting in lower scores for withdrawal symptoms. Finally, both MVPA and mobile phone dependence among adolescents were assessed using questionnaires, which are subject to recall bias inherent in self-report measures.

### 4.3 Adolescents who meet the open-skill MVPA guidelines have lower mobile phone dependence

Firstly, in the stratified analysis of open-skill activities, adolescents who met the MVPA guidelines scored significantly lower on withdrawal symptoms than those who did not meet the guidelines, while no significant differences were found in the dimensions of craving and physical and mental impacts. This result suggests that open-skill MVPA is negatively associated with withdrawal symptoms of mobile phone dependence. The cognitive activation induced by open-skill PA generates a synergistic effect with the behavioral substitution and emotional regulation functions of adequate MVPA. On the one hand, continuous movement adjustment in dynamic environments exercises cognitive control ability and enhances adolescents’ tolerance to withdrawal responses (47). On the other hand, sufficient MVPA further reduces mobile phone usage time and relieves withdrawal discomfort through physiological emotional improvement (23, 48).

Secondly, unlike open-skill activities, adolescents meeting closed MVPA guidelines showed no significant differences from those who failed to meet the guidelines in all three dimensions of mobile phone dependence: withdrawal symptoms, craving, and physical and mental impacts. This indicates that closed-skill activities show no significant association with mobile phone dependence, even when performed at the recommended intensity and duration. The findings further highlight the critical role of exercise type. Characterized by standardization and repetition, closed activities cannot significantly activate cognitive function or provide sufficient psychological substitution value even with increased duration (16). Although adequate closed-skill MVPA can improve physical health, it fails to target the core cognitive and psychological mechanisms of mobile phone dependence, resulting in a weak correlation with its associated symptoms.

In addition, a set of strategies was formulated in this study to control the potential type I error (false positive) in subgroup analyses and ensure the reliability of research findings. First, all subgroup analyses were predefined without post-hoc exploratory grouping. All subgroup tests were prespecified prior to statistical analyses; subgroup stratified regression was performed solely based on one stratification variable, namely PA type (open-skill vs. closed-skill PA), with only two subgroups divided. This approach greatly reduced the total number of statistical tests and prevented excessive accumulation of tests and inflated false positive risk arising from unrestricted post-hoc subgroup splitting. Second, large and balanced samples mitigated false positives caused by random fluctuation. The total valid sample size was 971 participants, including 555 individuals in the open-skill PA subgroup and 416 in the closed-skill PA subgroup. The two subgroups featured sufficient and relatively balanced sample sizes. Large samples diminish spurious significant associations stemming from random variation, lower randomly generated positive findings, and alleviate type I error inflation. Third, a consistent and strict significance level of *α* = 0.05 was adopted throughout the study. The significance threshold was not arbitrarily loosened for distinct models or subgroups, thereby uniformly constraining the overall false positive risk. Finally, identical confounding variables were adjusted across all models to reduce spurious associations induced by confounding bias. All stratified subgroup regression models were adjusted for age, gender, residential area, BMI, and SES. Such adjustment eliminated spurious associations caused by confounders, prevented confounding effects from being misinterpreted as genuine associations between variables, and indirectly decreased false positive conclusions.

### 4.4 Discussion on control variables

Firstly, age was significantly positively correlated with withdrawal symptoms, craving, and physical and mental impacts of mobile phone dependence. After entering junior school, adolescents experience a rapid increase in self-awareness and social needs, and mobile phones become a central tool for meeting social, entertainment, and information-seeking needs (49). Meanwhile, academic pressure gradually rises with grade level, and some adolescents use mobile phones to cope with stress, thereby exacerbating dependent behaviors (41, 42).

Secondly, males score significantly higher than females across all three dimensions of mobile phone dependence. In terms of behavioral characteristics, males tend to prefer highly stimulating content such as mobile games and short-video clips, which renders them more prone to impulsive usage and psychological cravings, accompanied by more severe withdrawal symptoms upon cessation of phone use. By contrast, females mainly engage in social chatting and video/audio browsing with relatively moderate usage behaviors and a lower level of smartphone dependence.

Thirdly, the association between BMI and mobile phone dependence varies hierarchically by MVPA type. Among adolescents engaging in open-skill MVPA, BMI is significantly negatively correlated with withdrawal symptoms. Open-skill MVPA (e.g., basketball and soccer) features dynamic confrontation and teamwork (17). Youths with moderate BMI tend to participate more actively and gain superior exercise experience, which effectively replaces mobile phone use and alleviates withdrawal symptoms; by contrast, those with abnormal body weight face participation constraints and gain limited relief from dependence. For the closed-skill MVPA group, BMI is significantly positively correlated with craving and physical- mental impairments. Closed-skill MVPA consists of standardized, repetitive movements and holds little appeal for overweight or underweight adolescents (17). Such individuals are inclined toward sedentary lifestyles and rely on mobile phones for entertainment, exacerbating usage craving and adverse physical and mental outcomes.

Lastly, SES is significantly negatively correlated with the craving dimension of mobile phone dependence. Families with high SES generally provide adolescents with abundant offline recreational options, social opportunities and educational resources, reducing youngsters’ reliance on virtual entertainment via mobile phones. Meanwhile, high-SES parents tend to adopt scientific parenting styles and implement proper guidance and supervision on mobile phone use, which effectively lowers adolescents’ psychological craving for cellphones. In contrast, low-SES families lack sufficient offline activity resources; mobile phones serve as low-cost tools for entertainment and social interaction, thereby easily triggering adolescents’ usage cravings.

### 4.5 The theoretical and practical significance of this study

This study has the following theoretical value. Firstly, it enriches the research system on the association between PA and mobile phone dependence. By integrating the two core dimensions of MVPA type and duration, this study overcomes the limitations of previous research that only focused on a single activity duration or type. It systematically reveals the association between meeting the time requirements of open/closed MVPA and mobile phone dependence among adolescents, and improves the theoretical framework linking multi-dimensional PA characteristics to problem behaviors. Secondly, this study provides a theoretical basis for adolescent PA health guidelines. It differentiates the effectiveness of different types of PA, supplements the differentiated theoretical evidence for adolescent PA recommendation standards, promotes the theoretical shift from “emphasizing activity duration” to “considering both type and duration,” and offers a new perspective for future interdisciplinary research on exercise intervention and addiction prevention.

This study has the following practical value. Firstly, it provides an operable exercise intervention program for adolescent mobile phone dependence. This study clearly recommends the combined model of open PA (basketball, football, table tennis, etc.) plus meeting MVPA duration standards, which can be directly applied in school physical education, family guidance, and mental health education to form a low-cost and easy-to-promote non-pharmaceutical intervention strategy for mobile phone dependence. Secondly, this study helps schools optimize the design of physical education curricula and after-school exercise. Schools can increase the proportion of open PA and ensure daily MVPA duration to precisely reduce the physical and mental harm caused by mobile phone dependence and improve adolescents’ health literacy (50). Finally, this study helps parents and teachers understand that “PA type is more important than PA duration alone,” guiding adolescents to shift from sedentary mobile phone use to social and interactive PA, thereby improving physical fitness as well as psychological dependence and behavioral problems.

### 4.6 Limitations and prospects of this study

Although this study puts forward some new viewpoints, it still has the following limitations. Firstly, this study is a cross-sectional survey, which can only reveal the correlation between MVPA and mobile phone dependence among adolescents, but cannot determine the causal relationship between variables. It is difficult to judge whether PA improves mobile phone dependence or mobile phone dependence leads to insufficient PA. Secondly, the sampling design incurred selection bias and limited the external validity of this study. A mixed sampling strategy combining convenience sampling and random class sampling was adopted for the survey. Only eighth-grade students from urban junior high schools were recruited, while adolescents from county and township schools as well as those in other grade levels were excluded. The sample thus lacks sufficient representativeness, and the research findings cannot be directly generalized to adolescents across regions, urban–rural areas and all school stages nationwide. Thirdly, the grouping strategy for PA carries a risk of exposure misclassification. Participants were categorized into the open-skill PA group and closed-skill PA group based on the sport they spent the most weekly time engaging in. Although this classification aligns with the study’s aim of exploring the long-term effects of dominant activity types and conforms to standard categorization practices used in existing adolescent exercise intervention research, adolescents with comparable participation durations in both open- and closed-skill sports can only be assigned to a single group. This approach underestimates the true effects of mixed- sport participation and introduces bias from exposure misclassification. Fourthly, data on PA types, duration and mobile phone dependence are all collected by self-report method, which is susceptible to subjective recall bias and social desirability bias, and the measurement accuracy is lower than that of objective monitoring tools (such as accelerometers). Fifthly, only a limited set of confounding variables were adjusted for in this study, and several key potential confounders were not incorporated into the analytical models. The regression models only controlled for age, gender, residential area, BMI, and SES, while omitting variables including daily screen time, sleep duration, depressive and anxiety symptoms, and academic burden. These factors are capable of simultaneously affecting both the level of PA and the severity of mobile phone dependence; accordingly, the observed association between PA and mobile phone dependence may be distorted by these unadjusted confounders. Finally, this study only analyzes the direct association between PA types, duration and mobile phone dependence, but the internal influence mechanism is not deeply revealed.

Accordingly, this study puts forward the following research prospects. Firstly, future research can adopt research designs such as cohort tracking and randomized controlled intervention experiments to clarify the causal effects of PA types and duration on adolescent mobile phone dependence through long-term follow-up and standardized intervention, and verify the effectiveness and stability of the intervention program. Secondly, optimize the sampling scheme to improve the generalizability of research conclusions. Future studies can adopt stratified cluster random sampling and conduct multi-center, large-sample surveys covering primary and secondary schools in multiple urban and rural regions nationwide, with balanced recruitment of adolescents across all school stages. This approach can reduce selection bias caused by convenience sampling and enhance sample representativeness as well as the external generalizability of research findings. Thirdly, optimize the grouping criteria and measurement methods for PA to reduce measurement bias. On one hand, revise the sport classification framework by adding a subgroup for participants engaging in mixed sports to mitigate exposure misclassification. On the other hand, establish a hybrid measurement design integrating objective monitoring devices and self-reported questionnaires. A subset of participants will wear accelerometers to calibrate self- reported PA data, and questionnaire items concerning activity contexts will be refined to lower recall bias and improve the accuracy of MVPA data. Fourthly, expand the range of confounding variables and refine the covariate adjustment framework. Future research should incorporate key confounders such as screen time, sleep status, depressive and anxiety symptoms, and academic stress into regression models to fully eliminate confounding bias that distorts the observed associations between variables and improve the reliability of study results. Fifthly, it is suggested to introduce mediation and moderation effect analysis to systematically explore the mediating role of executive function, social support, emotional experience and so on between PA and mobile phone dependence, as well as the moderating role of gender, family parenting style and so on, so as to improve the theoretical interpretation framework. Finally, based on the conclusions of this study, it is suggested to build standardized and feasible PA intervention programs for schools, families and communities, focusing on the promotion of open-skill sports, so as to provide scientific and practical guidance for the prevention and intervention of mobile phone dependence among adolescents.

## 5 Conclusion

This study explored the correlation between the type and duration of MVPA and adolescent mobile phone dependence, and drew the following conclusions. The results showed that open-skill MVPA was significantly negatively correlated with the physical and mental impacts caused by mobile phone dependence among adolescents. Meeting the daily recommended MVPA duration was significantly positively correlated with withdrawal symptoms of mobile phone dependence; however, its combined effect with open-skill MVPA was significantly negatively correlated with withdrawal symptoms. In contrast, closed-skill MVPA showed no significant correlation with any dimension of mobile phone dependence, regardless of whether the recommended duration was met. This study enriches the theoretical framework regarding the association between multi-dimensional PA characteristics and adolescent mobile phone dependence, identifies the combined effect of PA type and duration, and provides a new theoretical perspective for using PA to intervene in addictive behaviors. In practice, the results can inform the optimization of school physical education curricula and the development of targeted PA guidelines for families and communities, helping to reduce mobile phone dependence and improve physical and mental health among adolescents by promoting open-skill sports and ensuring adequate MVPA duration.

## Data Availability

The raw data supporting the conclusions of this article will be made available by the authors, without undue reservation.
